# HIV prevention intervention for substance users: a review of the literature

**DOI:** 10.1186/s13011-018-0189-7

**Published:** 2019-01-03

**Authors:** Adel Elkbuli, Valerie Polcz, Brianna Dowd, Mark McKenney, Guillermo Prado

**Affiliations:** 1 Department of Public Health Sciences, University of Miami Miller School of Medicine, Miami, Florida, USA; 2 Department of Surgery, Kendall Regional Medical Center, Miami, Florida, USA; 3 Department of Surgery, University of South Florida, Tampa, Florida, USA; 4 Florida International University, Miami, Florida USA

**Keywords:** Drug use disorder, HIV/AIDS, Injection drug users, Behavioral interventions, Intervention effectiveness, Sexual behavior, Universal HIV prevention interventions

## Abstract

**Background:**

Behavioral Interventions are needed to prevent HIV in substance users, which is associated with higher risk for contracting HIV via unprotected sexual intercourse or syringe-based exposure. We reviewed universal HIV prevention interventions targeting intravenous drug users (IDUs) and non-IDUs (NIDUs) to identify which prevention interventions are the most effective at reducing HIV transmission risk among IDU’s and NIDU’s and identify gaps in the literature.

**Methods:**

A PubMed literature review (1998–2017), limiting studies to universal HIV prevention interventions targeting adult HIV-negative substance users. Interventions were compared across sample sizes, sociodemographic, intervention setting, study design, use of theoretical models, and intervention effects.

**Results:**

Of 1455 studies identified, 19 targeted IDUs (*n* = 9) and NIDUs (*n* = 10). Both IDU and NIDU studies were conducted in substance use treatment centers and included both group (44% vs. 73%) and individual-based (56% vs. 27%) methods; only one NIDU study used a couple-based intervention. All IDU, and 89% of NIDU, studies used explanatory and behavior-change theoretical models to guide selection of intervention mechanisms. Reduction in frequency of risky sexual behaviors were observed in 33% IDU and 64% NIDU studies, where 56% of IDU studies effectively increased drug use-related hygiene and 67% decreased frequency of injections. Eight studies included start-of-study HIV testing and five examined HIV seroconversion.

**Conclusion:**

The interventions reviewed demonstrate promising results for decreasing risky sexual practices for NIDUs and reducing high-risk drug practices for IDUs, thereby reducing HIV transmission risk. Future studies should include HIV testing and measurement of HIV seroconversion to fully elucidate intervention effects.

## Introduction

In the era of highly active antiretroviral therapy (HAART), the incidence of new human immunodeficiency virus (HIV) diagnoses continues to remain high, with certain sociodemographic groups experiencing increased rates of HIV compared to the general population. Substance users in particular are at substantially increased risk of contracting HIV. The Substance Abuse and Mental Health Services Administration (SAMHSA) reports that approximately 81% of individuals living with HIV have used illicit substances at least once in their lifetime, with approximately 17% of HIV-positive individuals having used injectable drugs during their lifetime [1]. Sharing of needles and unprotected sexual contact are two high-risk behaviors that increase HIV transmission among substance users. Most individuals who contract HIV do so through unprotected sex, putting substance users at increased risk due to disinhibition as a result of intoxication, as well as through trading sexual favors for drugs [2].

Substance users belonging to marginalized sociodemographic groups are also at greater risk for contracting HIV. The Centers for Disease Control Drug Surveillance Report (2011–2016) indicated that of all injection drug users sampled, there were greater proportions of African-American (41.2%) HIV-positive injection drug users than white (32.5%) injection drug users [3]. Additionally, Hispanic injection drug users have a significantly higher estimated rate of HIV infection as compared to white non-Hispanic injection drug users, 4.9 per 100,000 people in the Hispanic population versus 0.9 per 100,000, respectively [4]. In addition, men who have sex with men (MSM) are at increased risk of substance-use related HIV infection, with 53% of substance use-related HIV cases comprised of MSM [1]. Reasons for the increased risk of contracting HIV in these particular sociodemographic groups may include lack of HIV and substance use education, lack of access to healthcare, discrimination, and increased stigma [2].

Furthermore, intravenous drug user (IDU) populations have different prevention needs than non-intravenous drug user (NIDU) populations. Some have reported that IDU populations require increased HIV testing and implementation of alternative programs to reduce sexual and drug use risk behaviors [5]. IDU populations also have higher transmission rates of HIV than NIDU populations due to widespread needle sharing practices, high rate of new injector initiation, and unsafe syringe cleaning practices [6, 7]. MSM who are also in the IDU population further have been reported to have increased violence, which should be considered in HIV prevention efforts [8]. Therefore, the IDU and NIDU populations should be separated to clearly define the best intervention methods for these differing groups at risk of contracting HIV.

Given the increased risk of HIV infection in substance users, universal interventions are needed to approach risk reduction. Universal interventions meaning prevention intervention efforts designed to reach the entire population of substance users rather than target specific subgroups of the population and focusing primarily on prevention of those who are not already HIV positive. Universal HIV prevention interventions including HIV education, drug use practices, and high-risk sexual practices may target multiple factors that contribute to increased risk among substance users. Although recent research showed that combined biomedical and behavioral approaches have the most potent effect on HIV risk reduction [9], the intervention topics that confer the greatest reductions in HIV risk have yet to be determined. Furthermore, researchers have not yet determined how best to target and deliver interventions to sociodemographic groups at the greatest risk for substance use-associated HIV. Intervention delivery methods, such as group vs. individual interventions, may significantly impact the effectiveness of interventions aimed at HIV prevention for those who need them most. Finally, there has not been a widely disseminated universal intervention technique for preventing HIV in substance users. Though many HIV prevention interventions are evidence-based, more work is needed to examine which of these interventions is the most effective [10, 11].

### Current study

The objective of our current study is to provide a review of behavioral HIV prevention interventions specifically targeted to substance users. We reviewed the literature regarding universal HIV prevention interventions in both intravenous drug users and non-intravenous drug users to identify interventions most effective at reducing HIV risk, as well as to identify any pertinent limitations or gaps in the literature. Our review aims to highlight intervention models, which may be useful in the development and adoption of new interventions on a greater scale. In the current study, we review the sample characteristics, intervention settings, theoretical backgrounds, methods, and effects of HIV prevention interventions for substance users, as well as potential mediators of intervention effects.

## Methods

We conducted a literature search for evidence-based universal HIV prevention interventions using PubMed to search the MEDLINE database (1998–2017). The PubMed search engine was chosen because PubMed has the MeSH vocabulary tool which provides a robust method of narrowing results [12, 13]. Further, PubMed is a human-curated database, which means articles are selected for inclusion by based on scholarly and quality criteria by literature review committees. PubMed’s accurate retrieval indicates that search results are reproducible and reportable. The target in this search was studies that addressed HIV prevention through an intervention that was targeted to those with substance use disorder who did not yet have HIV. To be included in the current review, the interventions had to meet all the following criteria:

1. The intervention should be focused on universal HIV prevention,
2. The intervention must be exclusively targeted to substance users,
3. The intervention sample was comprised of a majority HIV-negative substance users, ensuring only universal interventions were considered, and
4. The intervention sample must consist of adults ≥18 years old.

**Fig. 1:**
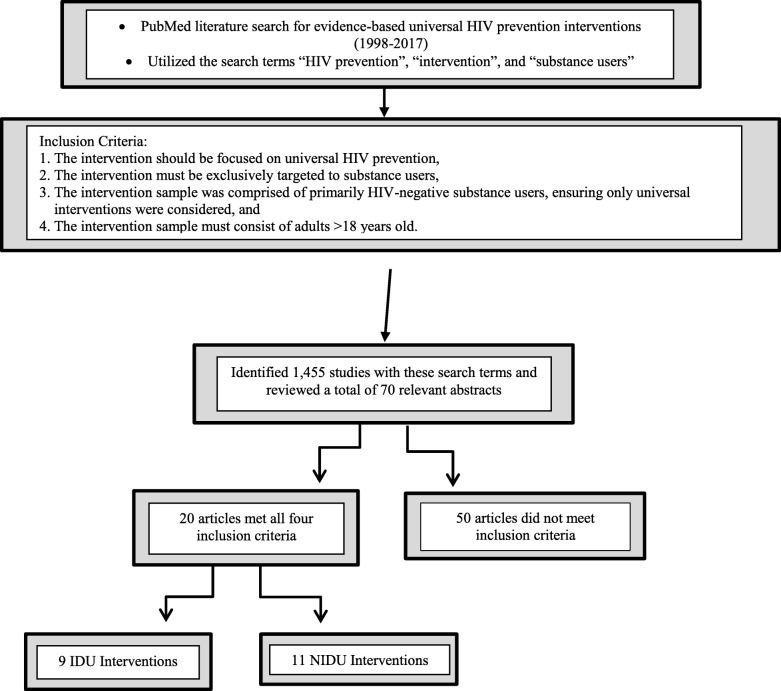
Flowchart shows the organization of the studies identified for the current review

We utilized the search terms “HIV prevention”, “intervention”, and “substance users” and did not apply any date, language, or publication status limitations in the searches. The search terms were systematically combined with “AND” statements. We identified 1455 studies with these search terms and reviewed a total of 70 seemingly relevant abstracts by analyzing each abstract for inclusion criteria, of which 19 met all four inclusion criteria. Any studies that did not meet all four inclusion criteria were excluded. The studies were screened by at least two authors and if all inclusion criteria were met, data on sample size, sociodemographic characteristics, intervention setting, intervention type, theoretical foundations, intervention length, and effects were extracted. We then divided the 19 studies into interventions which targeted IDUs (*n* = 9) and interventions which targeted primarily NIDUs (*n* = 10). Figure 1 shows a flowchart describing the organization of the studies identified for the current review. The primary outcome was improved HIV prevention interventions for IDU and NIDU populations in the future by the accumulation of current evidence.

## Results

### HIV prevention interventions for intravenous drug users (IDUs)

We identified nine HIV prevention interventions that targeted IDUs specifically [14–22]. These interventions were considered universal because the efforts were designed to eventually help the entire population. We examined and compared sample characteristics, intervention setting, theoretical background/approach, intervention methods, and intervention effects of these five universal HIV prevention studies below.

### Sample characteristics

The interventions targeted various at-risk IDU populations. The samples sizes of the IDU interventions ranged from 226 to 7132 participants. All universal IDU interventions included mostly young and/or middle-aged adults, with overall age ranges from 18 to 58, and with all studies reporting an average age of mid-to-late thirties. Two studies included a majority sample of heterosexual white male participants [15, 16]. One study included both genders with unspecified sociodemographic data [21]. Six studies included majority samples from sociodemographic minority populations [14, 17–20, 22]. Of the six studies that focused on sociodemographic minority populations, one targeted female sex workers [18]. Two included mostly African-American participants of both genders [17, 19]. Two included relatively similar proportions of white, African-American, and Latino participants of both genders [14, 22]. One included entirely male, Asian participants [20].

### Intervention setting

The intervention settings ranged dramatically between studies. Two of the studies obtained their participants from substance-use treatment facilities: one of these studies obtained participants from a substance use disorder treatment program through APT Foundation clinics in Connecticut [16], while the other enrolled participants from eight residential detoxification clinics [15]. Two interventions were conducted in medical offices [20, 22]. The other five interventions were conducted within community-based settings [14, 17–19, 21]. One study obtained their participants through non-governmental organization (NGO) outreach from various locations such as hotels, bars, brothels, street corners and alleys in Tijuana and Ciudad Juarez, Mexico [18]. Booth and colleagues [14] recruited their participants from eight U.S. cities that were a part of the National Institute on Drug Abuse (NIDA) Cooperative Agreement, with interventions taking place in community and project offices. Tobin and colleagues [17] recruited their participants via street-based outreach, word of mouth, advertisements and referrals from community agencies, with interventions conducted in a group setting within the community. Mihailovic and colleagues [19] recruited participants by street-based outreach, word of mouth, and advertisements posted throughout the community. Finally, Simmons and colleagues [21] recruited participants by outreach through social networks of PWID.

### Theoretical background and approach

Theoretical backgrounds and approaches for each of the nine IDU intervention studies varied greatly. Booth and colleagues [15] employed the use of the Counseling and Education (C&E) model where participants are provided with basic education on HIV/AIDS, instructed on how to reduce risk infection, and are tested for HIV. Participants also rehearse risk reduction techniques such as cleaning injection equipment and using a condom [15]. Counseling and education are provided both before and after HIV testing [15]. The goals of this model included education about the transmission of HIV and hepatitis C virus (HCV) along with learning and adopting behaviors that help to prevent transmission [15]. Simmons and colleagues [21] also utilized the Counseling and Education (C&E) model where participants were educated on the transmission of HIV and instructed on how to prevent HIV transmission. Further, Des Jarlais and colleagues [22] used another education model along with counseling in the intervention methods. The goal of these studies were to provide education on the dangers of HIV and provide services to reduce HIV transmission [21, 22]. By contrast, Copenhaver et al. [16] utilized an adapted version of an evidence-based intervention, Holistic Health Recovery Program (HHRP). The basis of the HHRP is the Information-Motivation-Behavioral Skills (IMB) model. The IMB model explains HIV-related behaviors and recognizes that information and motivation have direct effects on both behavioral skills and health behavior. Their goal was to design and implement the adapted version, Community-Friendly Health Recovery Program (CHRP), which is designed to reduce sex- and drug-related HIV risk behavior through group sessions into the substance use community-based organization, APT Foundation, Inc. [16].

The intervention by Vera and colleagues [18], Mujer Más Segura (Safe Women), incorporated various theories and models into four intervention arms that varied based on participant involvement with no control group. The first intervention arm consisted of didactic intervention only where the Counseling and Education (C&E) model was employed and involved basic education on HIV/AIDS as well as risk reduction education [18]. The second intervention arm consisted of interactive and didactic interventions on injection where participants were provided basic education on HIV and risk reduction, shown an educational video and rehearsed safe injection techniques [18]. The third intervention arm consisted of interactive and didactic interventions on sexual behavior where participants were provided basic education on HIV and risk reduction, participated in an educational discussion on safe sex, and rehearsed condom use techniques [18]. The final intervention arm consisted of a combination of interactive and didactic interventions on both injection and sexual behavior where the methods of the second and third intervention arms were combined [18]. Intervention groups utilized the fundamentals of Motivational Interviewing (MI), Social Cognitive Theory (SCT), and Theory of Reasoned Action (TRA) into the four intervention arms. These theories and models were obtained from highly efficacious evidence-based interventions, STRIVE (Study to Reduce Intravenous Exposures) and DUIT (Drug User Intervention Trial), which targeted HIV-negative IDUs [18].

Booth and colleagues [14] derived their intervention from large-scale HIV prevention efforts targeting IDUs. This effort was sponsored by NIDA, and the Cooperative Agreement (CA) for AIDS Community Based Outreach/Intervention initiated in 1990. While this universal intervention had been previously evaluated at individual sites, this study was the first to measure the impact of the CA intervention across multiple sites on a large scale [14].

Finally, Tobin and colleagues [17] utilized peer-based social network methods to recruit participants and facilitate their HIV prevention intervention. These social network methods are derived from social influence theories, which posit that individuals can be spheres of influence within their social networks. Tobin and colleagues first recruited IDUs, and then trained these IDUs to recruit members of their own social networks to participate in the intervention [17]. Mihailovic and colleagues [19] also utilized peer-based interventions by recruiting and training IDUs who then recruited other IDU’s within their community and social network. The goal of this model included providing information about HIV prevention and teaching participants the skills needed to promote risk reduction within their personal risk networks [19].

Goswami and colleagues [20] utilized the “Avahan” model, an India AIDS Initiative, supported by the Bill & Melinda Gates Foundation. This model was initiated in 2003 and focused on prevention programs and targeted interventions in what was considered to be a concentrated epidemic [20]. Integrated Behavioral and Biological Assessment (IBBA) was also utilized [20].

### Intervention methods

Intervention length and dose varied throughout the nine IDU interventions, however, each of the interventions were relatively short. Intervention length varied from 30 to 60 min per session, and number of sessions varied from one to seven sessions. Two interventions were group-based [16, 21]. Five were individual-based [14, 15, 18, 20, 22]. Two used both individual- and group-based methods [17, 19]. We present the individual characteristics of each intervention in Table 1.

**Table 1:** Characteristics of IDU and NIDU Interventions

	Sample Size	Sociodemographic Characteristics	Intervention Setting	Intervention Type	Theoretical Foundations	Dose/Length	Effects
IDU Interventions							
Copenhaver et al. (2007) [16]	226	51% Male; 68% Caucasian, 18% Black, 13% Hispanic, 1% American Indian	Substance use treatment clinic in CT. (APT Foundation, Inc.)	Group-based	Information-Motivation-Behavioral Skills model (IMB)	4 sessions; 50 min per session	Increased HIV risk reduction knowledge, safe sexual behavior knowledge, and motivational outcomes
Vera et al., (2012) [17]	584	100% female sex workers	Project offices and mobile units in Tijuana and Ciudad Juarez, Mexico	Individual	Social Cognitive Theory (SCT), and Theory of Reasoned Action (TRA).	1 session; 60 min	Null effects on sexual risk and drug outcomes for interventions vs. control
Booth et al. (1998) [14]	3743	71% Male; 37% Black, 34% White, 23% Latino & 6% Native American	Community and project offices	Individual	National AIDS Demonstration Research (NADR) program and Cooperative Agreement (CA) for AIDS Community Based Outreach/Intervention	2 sessions; length unspecified	Reduced drug injection
Booth et al. (2011) [15]	623	76% Male; 73% Caucasian, 8% African American, 10% multi-racial and 9% Latino/Hispanic ethnicity	Residential Detoxification Centers	Individual	Counseling and Education Model	Two 30 min sessions & one 45 min session	Decreased days injecting, use of unclean syringes, sharing cottons/cookers/rinse water and sharing the drug solution; but no differences between intervention groups
Tobin et al. (2011) [17]	227	60% male; 86% African-American,	Group setting within the community	Group-based and Individual	Social Influence Theory	7 sessions; length unspecified	Decreased use of unclean needle, cooker and cotton for injection and splitting drugs
Mihailovic et al. (2015) [19]	227	55% male; 85% African-American	Project office in community	Group-based and individual	Informational and counseling model	7 sessions over 18 months	Increased conversation about HIV prevention among substance users
Goswami et al. (2014) [20]	3349	100% male; 100% Asian	Medical clinic in two states of India	individual	Integrated Behavioral and Biological Assessment (IBBA)	2 rounds over 6 years	Increased safe injecting practices and safe sex behavior
Simmons et al. (2015) [21]	1123	73% male; 27% female	Project offices in Philadelphia and Chiang Mai	Group-based	Educational and counseling model	Session number unspecified; 30 months in length	Decreased injection risk behaviors and increased diffusion of HIV information
Des Jarlais et al. (2014) [22]	7132	82% male; 19% white; 33% African-American; 48% Hispanic	Beth Israel Medical Center in New York	individual	Educational and counseling model	1 session; unspecified length	Mostly null effects; decreased unprotected sex
NIDU Interventions							
Nydegger et al. (2013) [28]	143	66% male; 45% Hispanic	Court-mandated outpatient drug education classes	Group-based	Implementation Intentions model	1 session; 60 min	Increased condom use implementation intentions
Tross et al. (2008) [30]	384	100% female; 58% white, 24% African-American	Substance use treatment facility	Group-based	Safer Sex Skills Building (SSB) model	5 sessions; 90 min per session	Decreased unprotected vaginal or anal sex occasions
Calsyn et al. (2013) [23]	66	100% male; 42% African-American; 27% Hispanic; 18% white	Substance use treatment facility	Group-based	Information-Motivation-Behavioral Skills (IMB) model	5 sessions; 90 min per session	Decreased frequency of unprotected sex; reduced number of sexual partners
Kurtz et al. (2013) [31]	515	100% MSM; 48% white; 26% Hispanic; 21% African-American	Academic--Field offices (2) in South Florida	Group-based and individual	Psychological Empowerment Theory	4 sessions; 120 min per session	No differences in sexual risk or drug risk behavior between intervention groups
Mansergh et al. (2010) [24]	1686	100% MSM; 40% white; 31% African-American; 19% Hispanic	Health Centers	Group-based	Cognitive Behavioral model (CBT)	6 sessions; 120 min per session	Decreased frequency of unprotected sex; reductions in sex while using drugs
McMahon et al. (2001) [25]	149	100% male; 59% African-American; 33% white	Substance use treatment facility (VA)	Group-based	Cognitive Behavioral Model (CBT)	4 sessions; 120 min per session	Mostly null effects; increased unprotected sex in the intervention group
McMahon et al. (2013) [26]	660 (330 couples)	50% Male; 50% Female; *Women only:* 52% Hispanic, 34% African-American	Academic--Field office in South Bronx	Couple-based & individual	NIDA Community-Based Outreach model; Social- Cognitive Theory, Information-Motivation-Behavior Skills model, Stages-of-Change model, Theory of Gender and Power	2 sessions; length unspecified	Reduced frequency of unprotected sex; reduced numbers of sexual partners; Reduced HIV incidence
Mimiaga et al. (2012) [27]	16	100% MSM; 62.5% white	Health Center—Fenway Institute, Fenway Health, in Boston, MA.	Individual	Behavioral Activation (BA) model	10 sessions; 50-min per session	Reduction in frequency of unprotected sex; reductions in frequency of sex while using drugs
Herrmann et al. (2013) [29]	56	71.5% male; 85.5% white	Academic--Substance use treatment trials	Individual	Not specified	1 session; 50 min	Increased HIV knowledge
Surratt et al. (2014) [32]	597	100% female; 100% African-American	Project field office in Miami	Group-based and individual	Strengths-based case management (SBCM) with Professional-Only (PO) or Professional-Peer (PP)	5 sessions over 8 weeks	Decreased HIV risk behavior and increased service utilization outcomes

Intervention methods varied based on theoretical approach, intervention goals, and target population. All nine of the IDU interventions used some form of HIV education [14–22]. Four studies used didactic condom use training (e.g. behavioral skills development using anatomical models) [14, 15, 18, 20], and three used interactive condom use training (group discussion, negotiation of condom use with sexual partner, development of risk reduction strategies, etc.) to reduce sexual risk behavior associated with HIV [16–18]. All nine universal interventions used drug use behavioral skills training, which included education ranging from how to bleach and clean needles to how to split drugs safely [14–22]. Six of the studies reviewed utilized HIV testing and counseling, which may reduce HIV infection through knowledge of status and serosorting [14, 15, 18–20, 22]. Interestingly, while five of the interventions recruited substantial numbers of sociodemographic minority participants, no IDU study described using specific cultural tailoring methods to enhance the intervention. We present intervention methods in Table 2.

**Table 2:** Methods Used in IDU and NIDU Interventions

	HIV education	HIV testing and counseling	Didactic Condom Use Training	Interactive Condom Use Training	Safer Drug Use Practices	Drug Use Reduction or Abstinence
IDU Interventions						
Copenhaver et al. (2007) [16]	X			X	X	X
Vera et al. (2012) [17]	X	X	X	X	X	
Booth et al. (1998) [14]	X	X	X		X	
Booth et al. (2011) [15]	X	X	X		X	
Tobin et al. (2011) [17]	X			X	X	
Mihailovic et al. (2015) [19]	X	X			X	X
Goswami et al. (2014) [20]	X	X	X		X	
Simmons et al. (2015) [21]	X				X	
Des Jarlais et al. (2014) [22]	X	X			X	X
NIDU Interventions						
Nydegger et al. (2013) [28]	X		X			
Tross et al. (2008) [30]	X			X		
Calsyn et al. (2013) [23]	X			X		
Kurtz et al. (2013)	X			X	X	X
Mansergh et al. (2010) [24]	X			X		X
McMahon et al. (2001) [25]	X			X	X	X
McMahon et al. (2013) [26]	X	X		X	X	X
Mimiaga et al. (2012) [27]	X			X	X	X
Herrmann et al. (2013) [29]	X					
Surratt et al. (2014) [32]	X	X			X	X

### Intervention effects

Of the nine interventions reviewed, Tobin et al. described improvements in sexual risk behavior as measured by self-reported condom use, number of sexual partners, and exchanging sex for money or drugs [17]. Two of the nine interventions reported intervention-related decreases in drug use [14, 17], with Tobin et al. reporting significant intervention-related decreases in risky drug use behavior (e.g., needle sharing, using unbleached needles, etc.) [17]. Mihailovic et al. described self-reported increased conversations about HIV prevention among substance users and their social network [19]. Finally, three interventions found significant increases in risky drug use knowledge, safe sexual behavior knowledge, and motivational outcomes using a pre-post design [16, 20, 21]. Interestingly, three studies found null effects of their interventions on reductions in risky drug use behavior in comparison with standard treatment control groups [15, 18, 22].

Only two of the nine studies measured potential mediators of intervention effects [15, 18]. Though the enhanced intervention examined by Booth and colleagues [15] did not show significant decreases in risky drug use behaviors as compared with a standard intervention, study results indicated that self-efficacy for safer injection practices was associated with decreases in risky drug use practices for the overall sample. Though Vera and colleagues [18] measured various possible mediators of intervention effects, including peer norms regarding injection, HIV knowledge, outcome expectancy, and the participants’ belief that they could practice condom use and safer injection. These researchers did not conduct mediation analyses, as their three intervention arms did not significantly differ from their didactic control group on any of their main study outcomes [18].

### HIV prevention interventions for non-intravenous drug users

We identified ten HIV prevention interventions that targeted NIDUs or mixed groups of drug users [23–32]. We examined the sample characteristics, intervention setting, theoretical background/approach, intervention methods, and intervention effects of these ten interventions in the following section.

### Sample characteristics

The NIDU interventions exhibited substantial variability in sample size and composition, ranging from 16 to 1686 participants of differing sociodemographic characteristics. Age ranges and means were relatively consistent throughout the ten NIDU studies, with ages ranging from young to middle-aged adults, and mean ages in the late thirties to early forties [23–32]. Five interventions were targeted to men only [23–25, 27, 31]. Three of these interventions were targeted to MSM specifically [24, 27, 31]. The remaining two studies targeted men of any sexual orientation [23, 25]. Of the other five interventions, two were comprised of primarily male samples [28, 29], one targeted heterosexual couples [26], and two targeted females [30, 32]. Seven studies used primarily ethnic minority samples [23, 26, 28–32], with ethnic minority groups comprised of mostly African-American and Hispanic participants. Three studies used primarily white samples [27, 29, 30].

### Intervention setting

Of the ten NIDU interventions, three were conducted within drug treatment programs [23, 25, 30], two were conducted within health centers [24, 27], three within academic settings [26, 29, 31], one conducted within court-mandated drug classes [28], and one conducted within the community [32]. Of those conducted within drug treatment programs, one was in an inpatient setting [25]. Two were community-based [23, 30]. Recruiting procedures for most of the studies were similar, with studies utilizing community outreach, flyers, and word of mouth [23–30, 32]. However, Hermann et al. recruited participants through other ongoing clinical trials [29], and one study used internet media to recruit participants [31].

### Theoretical background and approach

The universal interventions for studies targeting NIDUs varied greatly in theoretical foundation. Four studies targeted sexual risk behavior specifically. Of these, one study examined an intervention to increase implementation intentions (situation-linked action plans) to use condoms for drug offenders participating in court-mandated drug classes, citing previous research linking implementation intentions to increases in health behavior [28]. Tross et al. [30] tested an evidence-based HIV/STD safer sex skills building (SSB) intervention for female drug users that had shown efficacy in a previous trial among women in methadone maintenance treatment. Calsyn and colleagues [23] determined the acceptability and effectiveness of a Culturally Adapted version of Real Men Are Safe (REMAS-CA), an HIV prevention intervention for men in substance use disorder treatment. The RESMAS intervention is based on the Information-Motivation-Behavioral Change (IMB) Model. Finally, Surratt and colleagues [32] utilized professional and professional-peer model interventions for female sex workers who use drugs.

For the six NIDU interventions targeting various HIV risk outcomes, two interventions were based off the cognitive behavioral model (CBT) [24, 25]. Similarly, the intervention developed by Mimiaga and colleagues [27] utilizes behavioral activation, which emerged from a component analysis of CBT. The goal of behavioral activation is to increase environmental reinforcement and reduce punishment. By contrast, Kurtz and colleagues [31] utilized interventions that were based on empowerment theory. McMahon and colleagues [26] tested three randomly assigned intervention conditions: 1) Couple-Based HIV Counseling and Testing (CB-HIV-CT), 2) Women-only Relationship-focused HIV Counseling and Testing (WRF-HIV-CT), and 3) NIDA HIV-CT which was considered the standard or “control” intervention. The control intervention was based on the NIDA Community-Based Outreach Model, as was the CB-HIV-CT intervention. The WRF-HIV-CT intervention was informed by an integrated theory of HIV risk that incorporated elements of social-cognitive theory, information-motivation-behavior skills model, stages-of-change model, and the theory of gender and power. Herrmann and colleagues [29] did not employ the use of any theoretical models, and their intervention was primarily didactic.

### Intervention methods

As previously mentioned, four of the ten NIDU interventions focused on exclusively reducing rates of high-risk sexual behavior [23, 28, 30, 32]. Of these four interventions, the intervention lengths varied from 60 to 90 min, and the intervention dose varied from one to five sessions [23, 28, 30, 32]. All four of these interventions were group-based and all included some form of HIV education. Two of the four interventions used interactive condom use skills training and condom use negotiation skills building [23, 30], with Nydegger et al. [28] utilizing didactic condom use training. Only one of these interventions utilized cultural tailoring to enhance intervention effects [23].

Six of the ten NIDU interventions focused on drug-related and other types of HIV risk outcomes in addition to sexual risk outcomes [24–27, 29, 31]. Of these six interventions, intervention lengths varied from 50 to 120 min, and doses varied from one to ten sessions [24–27, 29, 31]. Two of the six interventions involved group-based sessions [24, 25], two were individual-based [27, 29], one used combined individual and group methods [31] and one was couples-based [26]. All six of these interventions included some form of HIV education [24–27, 29, 31, 32]. Five of the six interventions focused on interactive behavioral skills building with regards to risky sexual behavior and drug use, including condom use skills, negotiating condom use, avoiding sex while using drugs, decreasing drug use, and/or safer drug use practices [24–27, 31]. One of these interventions was primarily didactic and focused mostly on HIV education [29]. Two of these interventions used HIV testing and counseling as part of the intervention [26, 32]. In addition, two interventions described some form of cultural tailoring for the intervention [25, 31]. NIDU intervention characteristics are presented in Table 1, and NIDU intervention methods are presented in Table 2.

### Intervention effects

Of the ten NIDU interventions, five reported intervention- related decreases in frequency of unprotected sex [23, 24, 26, 27, 30]. Two interventions reported decreases in numbers of sexual partners [23, 26]. In addition, two studies reported reductions in the frequency of sex while using drugs [24, 27]. One study reported increases in condom-use implementation intentions [28]. One study reported increases in HIV knowledge [29]. Finally, two studies reported intervention-related decreases in HIV incidence [26, 32]. Interestingly, two studies reported either null or iatrogenic effects on their universal outcomes [25, 31]. Kurtz and colleagues [31] did not find significant differences between their control and intervention groups in sexual risk behavior or drug risk behaviors. McMahon and colleagues [25] reported primarily null findings as well. However, these researchers found intervention-related increases in unprotected sex which the authors report was predominantly attributable to initiation of sexual activity among a subgroup that had reported abstinence prior to intake, rather than to an increase in the number of partners or to decrease in condom use among those who were sexually active prior to intervention.

Three of the ten studies examined variables that could be potential mediators of intervention effects [25–27]. Mimiaga et al. [27] measured motivation to practice safer behavior and behavioral skills for HIV prevention. While these factors increased with the intervention, Mimiaga and colleagues did not conduct mediation analyses to evaluate the relationships between these variables and their universal outcomes; rather, these variables were measured as outcomes themselves [27]. McMahon and colleagues [25] measured factual HIV knowledge, perceived susceptibility to HIV, anxiety regarding acquiring HIV, and self-efficacy to practice safe sexual behavior. However, this study did not find any intervention-related changes in these variables and did not conduct mediation analyses [25]. Finally, McMahon and colleagues [26] conducted mediation analyses and determined that HIV infections were prevented through intervention-associated reductions in unprotected sex and drug risk behavior.

## Discussion

Our review of 19 HIV prevention interventions for both IDUs and NIDUs revealed the majority of interventions to have positive effects on reducing rates of new HIV infection. The majority of interventions demonstrated improvements in at least some of their HIV prevention outcomes (80%), with 45% demonstrating reductions in sexual risk behavior, and 40% demonstrating reductions in drug use or risky drug use behavior. Although the majority of both the IDU and NIDU interventions reported favorable intervention effects, two of the NIDU interventions and two of the IDU interventions reported null or iatrogenic intervention findings [18, 22, 25, 31].

The question of how most interventions achieved at least some overall risk reduction is more complex. Most of the intervention studies for both IDUs and NIDUs used some form of condom training and education to improve sexual risk outcomes, as well as some form of HIV education. In addition, the majority of the intervention studies focused on either decreasing drug use or high-risk drug using behavior, although these methods varied based on intervention outcomes and intervention target group. For example, many of the interventions targeted to IDUs reviewed needle cleaning and disposal practices, while interventions targeted to NIDUs more often focused on drug use during sexual activity.

Although there were some similarities in intervention methods, certain factors may have contributed to the success of the various interventions in mitigating HIV risk. To start, universal intervention setting may have impacted the results of each study, influencing the sample receiving the intervention. For example, some of the interventions examined in this review [16, 22, 23, 25, 29, 30] incorporated interventions into substance use disorder treatment programs—the samples in these programs may have exhibited more severe drug use than samples drawn from the community. When disseminating these interventions researchers should consider the setting the intervention was originally tested in and exercise caution when making inferences on the generalization of such interventions to substance users more broadly. Further, although most interventions demonstrated favorable effects, theoretical approach may have influenced the effectiveness of these interventions. Theoretical approaches varied significantly between studies, and more work is needed to determine which theoretical approaches yield the most efficacious interventions. Potentially, whether or not a particular intervention was culturally tailored may influence the effectiveness of the intervention. One of the interventions reviewed demonstrated that cultural tailoring augmented intervention effects in ethnic minority drug users, as compared with a previous study of the same intervention [23]. Further work is needed to determine the effects of cultural tailoring on intervention success. Generally, medical and psychological disorder comorbidity among subjects were not considered in the studies included in this review. Though, psychological disorder comorbidity could significantly affect the results of the intervention and should be considered an important factor in future research regarding HIV prevention interventions. Additionally, inclusion of a follow-up period would help to clarify the maintenance effect of interventions and risk reductions over time. Some studies included sessions that extended multiple years, but specific follow-up meetings would elucidate the results of the intervention. Future research should extend the follow-up period to provide clarification of the long-term effects of HIV prevention intervention. Ultimately, our review has identified many factors to be considered when formulating or disseminating a universal HIV prevention intervention for substance users.

It is noteworthy that effect size may impact the results of each study. Effect size in medical literature is the magnitude of the difference between groups [33]. Effect is often reported as a *p* value, however, this only demonstrates that an effect exists. The p value – indicating the effect is statistically significant – does not demonstrate the size of the effect nor does it necessarily suggest that the improvement is clinically meaningful. Effect size provides a scale-free measure that reflects the practical meaningfulness of the difference or the relationship among variables [34]. Therefore, the results of each study should report beyond statistical significance and attempt to examine the clinical impact of the results through effect size. This would considerably improve the quality of HIV prevention intervention research which might help to improve the clinical applicability of these HIV interventions.

Overall, HIV prevention interventions that seem most promising are those that incorporate theoretical bases such as the IMB model. For IDU prevention interventions, individual interventions or interventions that contain individual portions seem to have the most effects on prevention and understanding of HIV. For NIDU prevention interventions, group interventions seem to have the most effect on prevention and increased HIV knowledge. The use of theoretical bases and individual versus group prevention interventions should be further analyzed in forthcoming research efforts.

### Limitations

There are limitations in the interventions we reviewed. Firstly, many of the interventions were targeted to specific sociodemographic groups of drug users, such as ethnic minorities or men who have sex with men 14, 17–20, 23–28, 31, 32]. While these interventions targeted sociodemographic groups with high risk of contracting HIV, the specificity of these studies to these particular groups limits the generalizability of these interventions. More work is needed to elucidate whether the positive effects found in many of the interventions reviewed are generalizable to other sociodemographic groups. Furthermore, the majority (75%) of studies we reviewed did not describe cultural tailoring of their intervention to meet the needs of the particular sociodemographic groups targeted in the interventions. As one study demonstrated, cultural tailoring may increase favorable intervention effects [23]. Future studies should aim to customize HIV prevention interventions to reflect the needs of their community, especially when targeting minority populations. Finally, all but two studies reviewed did not measure HIV seroconversion as an outcome, but rather theoretical modifiers of HIV seroconversion such as risky drug and sexual behavior. Understandably, many of the studies were not powered to detect intervention effects on HIV seroconversion; however, understanding potential intervention effects on HIV seroconversion is important, as mediators such as sexual risk behavior may not perfectly relate to changes in HIV risk as a result of intervention. Larger and more highly powered studies are needed to examine the effects of universal HIV prevention interventions on HIV seroconversion in substance users.

Further, there are some limitations in this systematic review. It is possible that some studies were missed in the search strategy, such as unpublished articles or relevant articles missed by the search terms. Also, there may be publishing bias in the original research studies where only significant and positive results were published and this bias would be transferred to the review. Additionally, a limitation in this review is that only studies with intervention samples that consisted of adults ≥18 years old were included.

## Conclusion

The current review examined universal interventions for new HIV infection in substance users. The vast majority of interventions reviewed had favorable effects on HIV knowledge, behavioral skills, sexual risk behavior, and/or risky drug use behavior, which may all be mediators of HIV seroconversion. More research is needed to adapt these interventions to other sociodemographic groups in order to determine the applicability of these interventions across various populations. In addition, larger studies are needed to examine the influence of these interventions on HIV seroconversion.
